# Mpox in Nigeria: Perceptions and knowledge of the disease among critical stakeholders—Global public health consequences

**DOI:** 10.1371/journal.pone.0283571

**Published:** 2023-03-30

**Authors:** Olajoju J. Awoyomi, Emmanuel O. Njoga, Ishmael F. Jaja, Felix A. Oyeleye, Priscilla O. Awoyomi, Musawa A. Ibrahim, M. A. Saulawa, Haruna B. Galadima, Adekunle B. Rowaiye, Taiwo I. Olasoju, Jamila A. Idrisa, Folasade D. H. Olalere, Mary I. Olasoju, Oluwatosin H. Adisa, Veronica E. Adetunji, Osagie O. Idemudia, Ekene V. Ezenduka, James W. Oguttu

**Affiliations:** 1 Department of Veterinary Public Health and Preventive Medicine, College of Veterinary Medicine, Federal University of Agriculture, Abeokuta, Nigeria; 2 Department of Veterinary Public Health and Preventive Medicine, Faculty of Veterinary Medicine, University of Nigeria, Nsukka, Nigeria; 3 Department of Agriculture and Animal Health, Florida Campus, University of South Africa, Johannesburg, South Africa; 4 Department of Medicine and Surgery, College of Medicine, University of Ibadan, Ibadan, Nigeria; 5 Department of Veterinary Public Health and Preventive Medicine, Faculty of Veterinary Medicine, Usmanu Danfodiyo University, Sokoto, Nigeria; 6 Department of Veterinary Public Health and Preventive Medicine, Faculty of Veterinary Medicine, Bayero University Kano, Kano State, Nigeria; 7 Department of Veterinary Medicine, Faculty of Veterinary Medicine, University of Maiduguri, Maiduguri, Nigeria; 8 Depatment of Agricultural Biotechnology, National Biotechnology Development Agency, Abuja, Nigeria; 9 Federal Ministry of Agriculture and Rural Development, Department of Veterinary and Pest Control Services, Epidemiology Division, Garki, Abuja, Nigeria; 10 Department of Medicine, College of Medical Sciences, University of Maiduguri Teaching Hospital, Maiduguri, Nigeria; 11 Department of Internal Medicine, College of Medical Sciences, University of Maiduguri Teaching Hospital, Maiduguri, Nigeria; 12 Lagos State University College of Medicine/Lagos State University Teaching Hospital, Ikeja, Lagos, Nigeria; 13 Department of Family Medicine, Sacred Heart Hospital, Lantoro, Abeokuta, Nigeria; 14 Department of Veterinary Public Health and Preventive Medicine, Faculty of Veterinary Medicine, University of Ibadan, Ibadan, Nigeria; 15 Ministry of Agriculture and Food Safety, Edo State, Nigeria; Universitas Syiah Kuala, INDONESIA

## Abstract

**Background:**

The mpox (monkeypox) disease is a re-emerging viral zoonosis of international concern that is endemic in parts of Africa. The mpox virus (MPXV), which was hitherto largely limited to some Central and West African countries, was declared a public health emergency of international concern by the WHO on July 23, 2022 following the rapid spread of the virus to non-endemic countries. Globally, as of March 16, 2023, the WHO had reported 86,496 laboratory-confirmed cases of mpox and 111 deaths in 110 countries. Of the 1,420 cases of mpox reported in Africa as of March 16, 2023, Nigeria alone recorded 57.1% (812) of the confirmed cases and eight fatalities recorded in the continent. To help improve on the understanding of the current situation in Nigeria, the present study assessed the perception and knowledge of mpox among Nigerian healthcare workers, academics and tertiary students. The study also sought to highlight the global public health significance of the MPXV, and recommend a One Health approach to limit exporting of the virus beyond the borders of Nigeria.

**Methods:**

A web-based cross-sectional survey was conducted between 24 July 2022 and 12 August 2022 to evaluate the perception and knowledge of mpox among 1544 Nigerians, consisted of healthcare workers (n = 832), academics (n = 306) and tertiary students (n = 462). Data on the respondents’ socio demographics and their information sources on mpox were also collected. Each correct response was allotted one point while an incorrect response was scored zero. The scores for perception and knowledge were dichotomized into positive (>5.5) and negative (≤5.5) and adequate (>5.8) and inadequate (≤5.8), respectively; using the average scores for perception and knowledge. The average score for perception and knowledge were summarised and presented as the mean and standard deviation (SD). Chi-square tests of association and binary logistic regression were carried out to determine factors associated with the outcome variables.

**Results:**

Of the 1452 respondents that had heard of mpox, 878 (60.5%) and 419 (28.9%) had adequate knowledge and positive perception concerning MPXV infection respectively. Average perception score was 5.5. Mean perception and knowledge scores were 4.5(SD: 2.0) and 5.8 (SD: 1.9), respectively. Factors that were significantly associated with knowledge level were age (p = 0.020) educational qualification attained (p = 0.004), occupation (p<0.001), and geopolitical zone of residency (p = 0.001). There was a positive correlation between perception and knowledge scores (r = 0.4, p<0.001). Positive perceptions were likely among respondents who had tertiary education, and residing in North-west Nigeria. Likewise, adequate knowledge scores were likely among respondents under 30 years of age, with tertiary education or reside in North-west Nigeria. Sources of information were significantly associated with perception (p = 0.004) and knowledge (p<0.001) of the respondents.

**Conclusion:**

The findings of this study show that there is disparity in the knowledge and perception of mpox in the study population, and as a result, there is a need to intensify awareness about MPXV infection to enhance positive perception among the respondents. This has potential to safeguard public health and contain the disease thus preventing it from spreading to the global community. A One Health approach involving animal and human health workers is imperative for improved knowledge and a good perception towards the disease among respondents, and enhanced active surveillance and early detection of MPXV in reservoir hosts (rodents and non-human primates); to prevent reverse zoonotic transmission of the virus at the human-animal interface.

## Introduction

Mpox is a re-emerging viral zoonosis caused by the mpox virus (MPXV), belonging to the genus *Orthopoxvirus*, and the family *Poxviridae* [1, 2]. Among the 10 species of viruses that constitute the genus, *Orthopoxvirus*, the MPXV and the smallpox viruses are zoonotic and hence the most important members of the genus from public health and One Health perspectives [1]. Phylogenetic evidences show that two separate genetic clades of MPXV exist: the Central African/Congo Basin (MPXV-ZAI-V79) and the West African (MPXV-COP-58) clades; with the former being more transmissible and virulent [3, 4]. However, in most mpox endemic countries of Africa, infections with both virus variants have been reported [5].

The MPXV infection was first diagnosed in 1958 from captive monkeys in Copenhagen, Denmark; hence the name “monkeypox”, which is now designated mpox [6, 7]. Later on in 1970 and 1971, human cases of MPXV infections were reported in children from the Democratic Republic of Congo (DRC) and Nigeria, respectively [2, 7]. In 2017, Nigeria witnessed a major outbreak of mpox during which 500 suspected cases and 200 laboratory confirmed case, with a case fatality rate of 6% was reported, mostly in young adult population [8, 9]. Since the recent outbreak of mpox that started in May 2022, 1,420 laboratory confirmed cases had been reported in Africa as of March 16, 2023, Nigeria has recorded 812 confirmed cases representing 57.1% of the cases in Africa [10–12]. Of the 812 confirmed cases in Nigeria, 726 (89.4%) were males and while 86 (10.6%) were females [10–12]. Nigeria has so far reported eight deaths from the 2022 outbreak [10–12]. However, mpox cases are believed to be grossly under-reported in Africa, especially in rural settings, due to limited medical facilities, self-medication, and poor disease reporting culture [13].

On the other hand, as of March16, 2023, 86, 496 confirmed cases of mpox and 111 deaths were reported globally across 110 countries/territories [11, 12]. Of these confirmed cases, 96.6% were recorded in male, with a median age of 34 years (interquartile range of 29 to 41 years) [11]. Based on sexual orientation, 89% of the confirmed cases were identified as being gay, bisexual and other men who have sex with men [11, 12]. Majority of the confirmed cases of mpox were reported from the American and European countries. Prior to this, cases of mpox had not been reported in these countries [11, 12]. Therefore, with the global escalation of the infection, the WHO [11] declared the 2022 MPX outbreak a Public Health Emergency of International Concern (PHEIC) on 23 July, 2022.

The preponderance of the 2022 mpox cases and fatalities occurring in traditionally non-endemic countries accorded the disease a global public health importance. In 2003, the first MPX outbreak outside Africa was reported in the US following contact with an infected dog, housed with Gambian pouched rats imported from Ghana [14, 15]. Although scientists are not certain about the actual reservoir hosts of mpox, rodents and non-human primates, including rope squirrels, tree squirrels, Gambian pouched rats, dormice, and *Macaca* species of monkeys are suspected to be the most probable reservoirs of the virus [16]. Zoonotic transmission of MPX may occur following direct contact with body fluids, cutaneous or mucosal lesions from infected animals. Reverse zoonotic transmission of MPXV from an infected dog owner has been reported [17]. Consumption of poorly-cooked meats or other edible animal products of infected animals is a major risk factor aiding the disease transmission. Human-to-human transmission of MPX may ensue following direct or indirect contact with cutaneous lesions, body fluids/secretions and respiratory droplets from infected persons. Vertical transmission of the virus in utero, via the placenta, from an infected mother to the foetus can occur [18]. Detection of MPXV DNA in seminal fluids and the preponderance of the 2022 mpox outbreak among gays, bisexuals, and other men having sex with men (MSM), particularly those with multiple sex-partners; suggests that the virus may be sexually transmitted [19–21]. However, the World Health Organisation (WHO) cautions against discrimination or stigmatization of patients on the bases of sexual orientation but encourages provision of care and health services, where possible [22].

Clinically, mpox is characterised by fever, intense headache, rash, back pain, myalgia, exanthema, asthenia, inflamed lymph nodes and other health complications [22]. Inguinal or cervical lymphadenopathy is a pathognomonic feature in mpox in contrast to other diseases that may present similar clinical syndrome [22]. The incubation period of mpox varies from five to 21 days depending on the infecting virus clade and the host’s immune status [6, 22]. Severe cases of mpox often occur among immune-deficient individuals, children and male adults but the severity usually depends on the extent/duration of exposure to the virus, the infecting virus clade, patient’s immune status and presence of other health complications [22].

The mpox is usually a self-limiting disease with symptoms lasting two to four weeks. Vaccination against smallpox was reported to be about 85% effective in preventing mpox [22, 23]. Recently, newer smallpox and mpox vaccine based on the modified vaccinia virus (Ankara strain) has been approved for vaccination against the disease in 2019, but the availability of the vaccine is very limited [22, 23]. Although there is no definitive cure for mpox, antiviral agents developed for the treatment of smallpox have been repurposed and licensed for the management of the disease [22]. These include cidofovir^®^, tecovirimat^®^ and vaccinia immune globulin which were granted emergency approval by the European Medicines Agency (EMA) for management of the 2022 MPXV outbreak in the EU [22, 23].

Cognisant of the fact that mpox is endemic in Nigeria [24]; it is not surprising that PCR confirmed cases of the disease are rising in the country. The rise in the numbers of cases in Nigeria has exceeded what has been reported in other African country, including the Democratic Republic Congo where the African index case was reported [11, 12]. Considering Nigeria’s vast human population estimated at 220 million as of March 2023 [25], there is need to understand the perceptions and knowledge of healthcare workers and other critical stakeholders in the health sector towards mpox. This is because the targeted population (healthcare workers, academics and tertiary students) are the “crème de la crème” in the society whose perceptions have the potential to shape the views/opinions of other people in the country, and this could either make or mar the disease containment measures. Results of the assessment of the perception and knowledge of important stakeholders with respect to mpox can be used to guide policy formulation for public health intervention towards preventing and controlling mpox in Nigeria. Curbing the spread of mpox in Nigeria, the most populous black nation, is imperative for controlling the disease not only in Africa but the rest of the world. This is appreciated when consideration is given to the fact that the MPXV has in the past been exported to other countries from Nigeria [14, 15]. Therefore, the study determined the perceptions and knowledge of Nigerian healthcare workers, academics and tertiary students concerning mpox, to make evidence-based recommendations for the prevention, control and possible eradication of MPXV infections in Nigeria. Furthermore, findings of the present study will contribute to improved understanding of how to limit case exportation and safeguard global public health.

## Methods

### The study area

**Fig 1:**
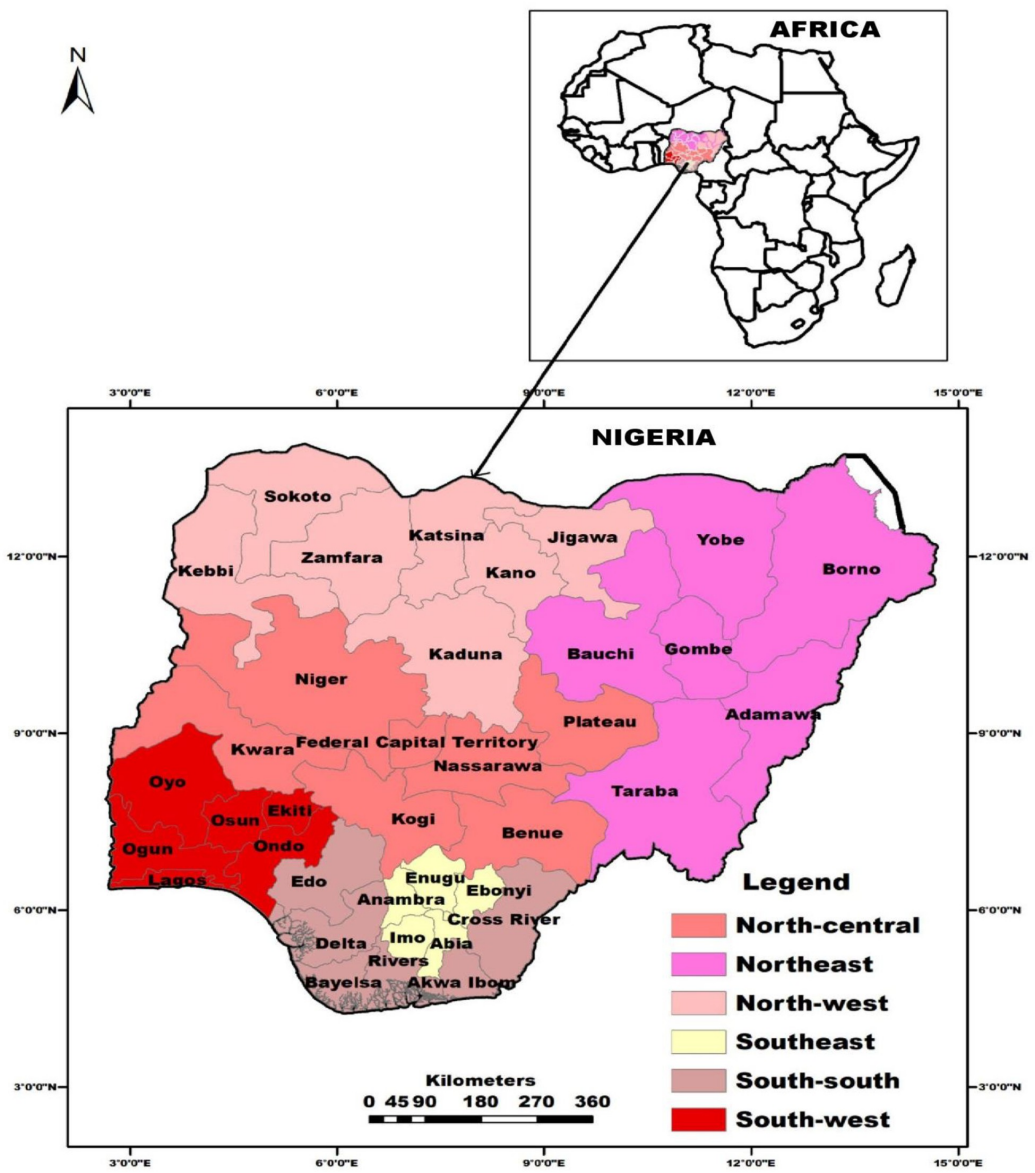
Map of Nigeria in Africa, showing country’s six geopolitical zones, their constituent states and the Federal Capital Territory, Abuja. ArcMap^®^ software version 10.2 (ESRI Inc., Redlands, CA, U.S.A.) was used to generate the maps while the shape-file was retrieved from DIVA- GIS (https://www.diva-gis.org/).

The survey was carried out in Nigeria, a West African country and the most populous black nation on earth. Nigeria has an estimated population of 220 million people [25] and is divided into six geopolitical zones—Southeast, South-south, South-west, Northeast, North-central and North-west (Fig 1). Nigeria is located in the Gulf of Guinea in Sub-Saharan Africa. The country has a total land mass of 923,769 Km^2^, a population density of 226 persons per Km^2^ and experiences a temperature range of 16° C to 45° C depending on the geopolitical zone and season of the year [26].

### Study design

**Fig 2:**
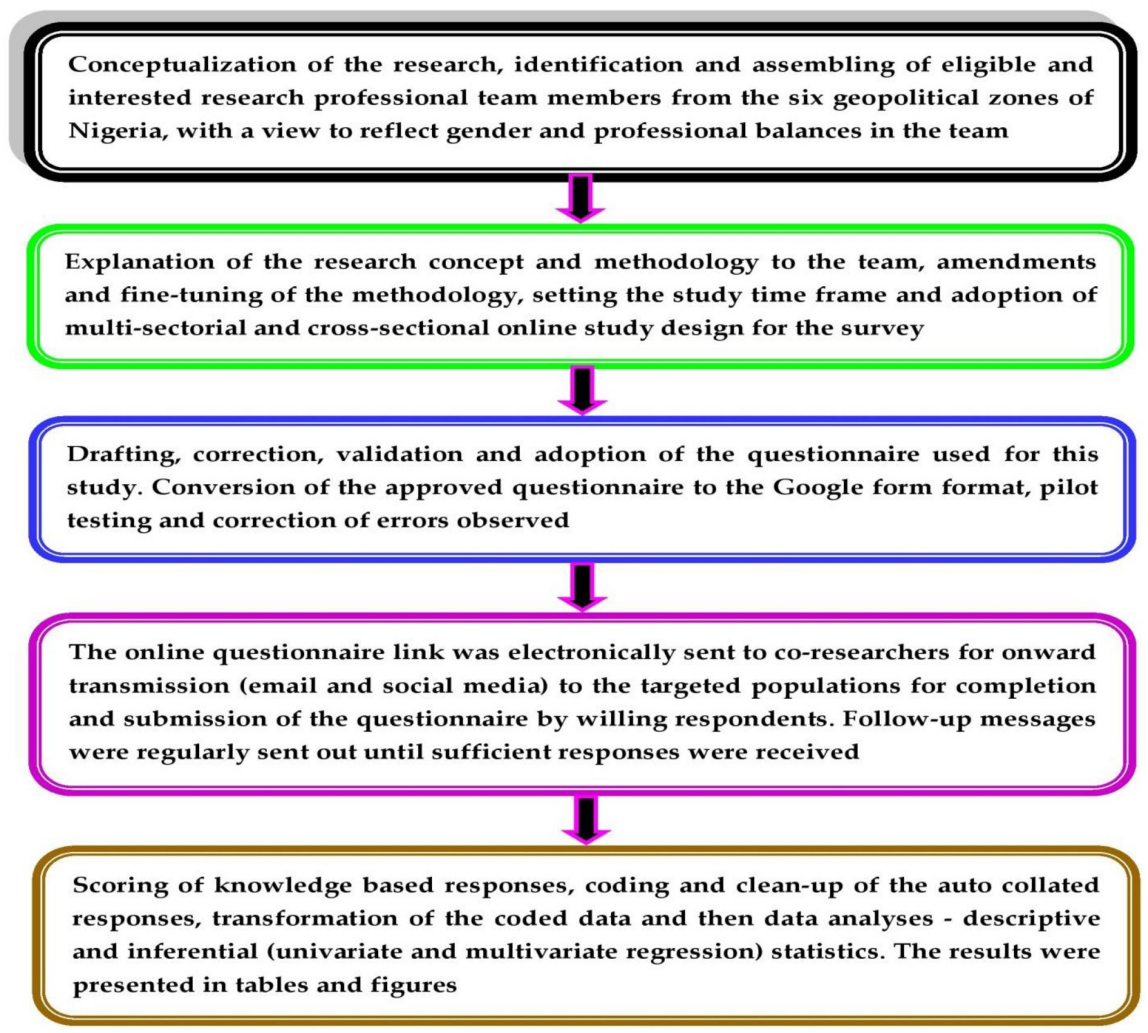
Schematic representations of the study design and methodology, from conceptualization to data analysis and presentation.

This online-based survey which targeted healthcare workers, academics and tertiary students adopted a cross-sectional study design. A minimum sample size (MSS) of 660 respondents was calculated using the Raosoft^®^ sample size calculator [27]. The MSS computation assumed an estimated target population size of 100,000, 5% error margin, 99% confidence level and 50% response rate. Although 660 was the MSS calculated, 1,544 respondents were surveyed for data accuracy. An overview of the study design and methodology, from conceptualization to the data analysis is schematically presented in Fig 2.

### Questionnaire development

A structured, validated and pilot-tested questionnaire with closed-ended questions prepared in Google form format (Alphabet Incorporated, California, USA), was used as the data collection instrument. The Google form consisted of 37 questions written in simple English and classified into four parts as follows: socio-demographics of respondents (seven questions), awareness of MPXV infection (two questions), perceptions of MPXV infection (11 questions) and knowledge of MPXV infection (17 questions). A copy of the questionnaire used in this study is available as a supplementary file (S1 Table).

Thereafter, the questionnaire was subjected to validation. First, we performed face and content validations, as described by Bolarinwa [28]. Additionally, the questionnaire was revised by a three-man panel of experts in viral zoonoses with profound experience in MPXV infection, from different geopolitical zones of Nigeria. The experts also assessed and scored each question based on relevance and clarity, using a 4-point scale, and made recommendations accordingly. From the scores, the scale-cumulative validity index (s-CVI) and the mean item-cumulative validity index (mean i-CVI) were computed according to the method previously described by Zamanzadeh et al. [29]. The calculated s-CVI and mean i-CVI for relevance were 0.84.and 0.93, respectively. Similarly, the calculated s-CVI and mean i-CVI values for clarity were 0.84 and 0.93, respectively. Thereafter, some questions were revised as recommended to enhance relevance and clarity. The questionnaire was pilot tested on 20 respondents before the actual survey, and errors noted were rectified. Responses from the pilot test were excluded from the result but were used to perform the Cronbach’s alpha test, to determine the reliability of the questionnaire. This yielded an alpha value of 0.751 (75.1% reliability).

### The survey procedure

This web-based survey in English was carried out following the Checklist for Reporting Results of Internet E-Surveys and techniques for conducting and reporting web-based studies [30]. Only respondents who have were ≥ 18 years old and are Nigerian-based healthcare workers, academics, or tertiary students were allowed to participate in the survey. To avoid duplicate or multiple responses, the settings of the Google Form were adjusted to enable a device (same internet protocol address) to submit a completed questionnaire only once. To ensure complete responses, answering the 37 questions was made compulsory but “I do not know” option was provided as appropriate.

The questionnaire link was sent to the targeted population via email and social media (Twitter, Facebook and WhatsApp). Follow-up messages were periodically sent to respondents who did not comply, until sufficient responses were received. At least 1,000 respondents; comprising of 500 healthcare workers and 250 academics and tertiary students each were targeted and invited to complete the questionnaire in each of the six geographical zones.

### Ethical approval and statement of informed consent

Institutional ethical approval (Ref No: VPHPM/UNN/23/011) to carry out this work was granted by the Research Ethics Committee of the Department of Veterinary Public Health and Preventive Medicine, University of Nigeria, Nsukka. Electronic informed consent to partake in the study was requested on the first page of the online questionnaire. Only respondents who willingly consented to participate in the survey by checking the “accept box” were permitted to complete and submit the questionnaire. Partaking in the survey was voluntary and at the discretion of the participants. Data privacy rights of the respondents were respected as their contact information (with the exception of their email addresses which were collected to prevent multiple responses), or personal identities were neither requested nor collected during the survey. The online-survey conformed entirely to the Helsinki declaration of the World Medical Association 2013 [31].

### Data management and analyses

The auto-collated responses from 1,544 successfully submitted online forms were cleaned up, coded and transformed for descriptive and inferential statistical analyses. Socio-demographic (gender, age, highest educational level attained, occupation, marital status, geopolitical zone of residence and locality of residence) data were descriptively (frequencies and percentages) analysed. Similarly, descriptive statistics were used to analyse variables on awareness and sources of information on mpox, and results presented as bar and pie charts, respectively.

To assess the perception of the respondents, 11 related questions were scored. Correct response to each question was scored one, while an incorrect response was scored zero. Maximum obtainable score for perception was 11. Knowledge score was assessed through responses to questions on symptoms, mode of transmission and control and prevention. The ability of the respondents to identify at least five symptoms and four routes of transmission were scored one each or else zero. Correct responses to questions (seven) on prevention and control were scored one each except the response to availability of vaccine which was not included. Maximum obtainable score for knowledge was nine. Continuous variables were presented as mean and standard deviation (SD), while categorical variables were summarised and presented as frequency or percentages. Both perception and knowledge scores were dichotomized into positive (> 5.5) and negative (≤ 5.5) and adequate (> 5.8) and inadequate (≤ 5.8), respectively using the average perception and knowledge scores as described by Alshahrani *et al*. [32]. The association between the respondents’ demographic characteristics and outcome variable (perception and knowledge levels) was assessed using Pearson chi-square test. All demographic characteristics of the respondents were included in the final binary logistic regression with eh exception of gender which had a p-value greater than 0.3 for both outcome variables (perception and knowledge levels). Hosmer-Lemeshow test was used to assess the model’s goodness fit of the final model. This yielded Chi-square and p-values of 4.2 and 0.835 for perception; and 13.9 and 0.085 for knowledge, respectively (S2 Table). The statistical significance was performed at 5% probability level using IBM^®^ SPSS statistics version 20 (SPSS Inc., Chicago, Illinois, USA). Statistical significance was accepted at p<0.05.

## Results

### Socio-demography and awareness of the respondents

**Fig 3:**
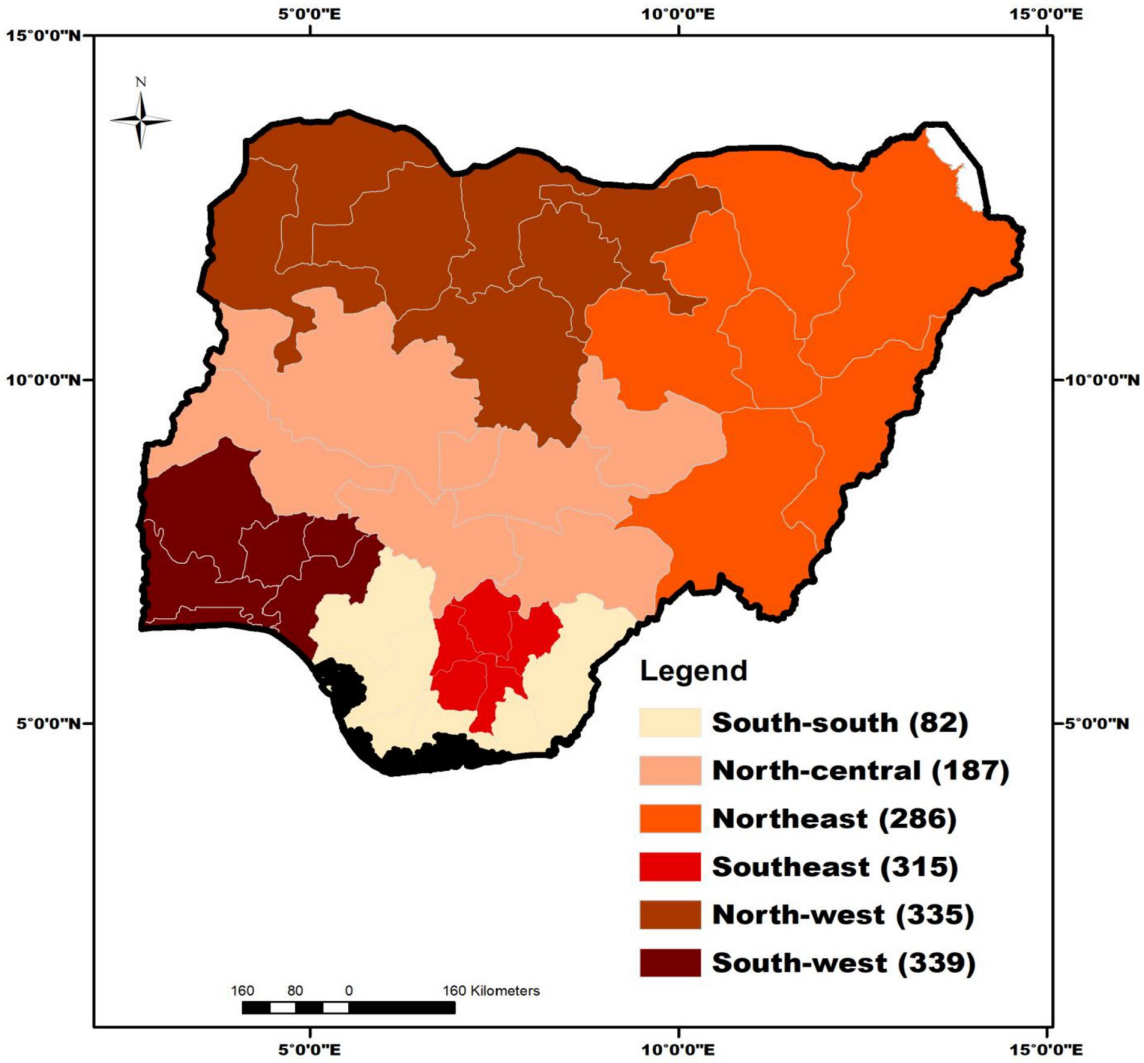
Distribution of respondents (n = 1544) surveyed for perceptions and knowledge about mpox across the six geopolitical zones of Nigeria. ArcMap^®^ software version 10.2 (ESRI Inc., Redlands, CA, U.S.A.) was used to generate the maps while the shape-file was retrieved from DIVA- GIS (https://www.diva-gis.org/).

**Fig 4:**
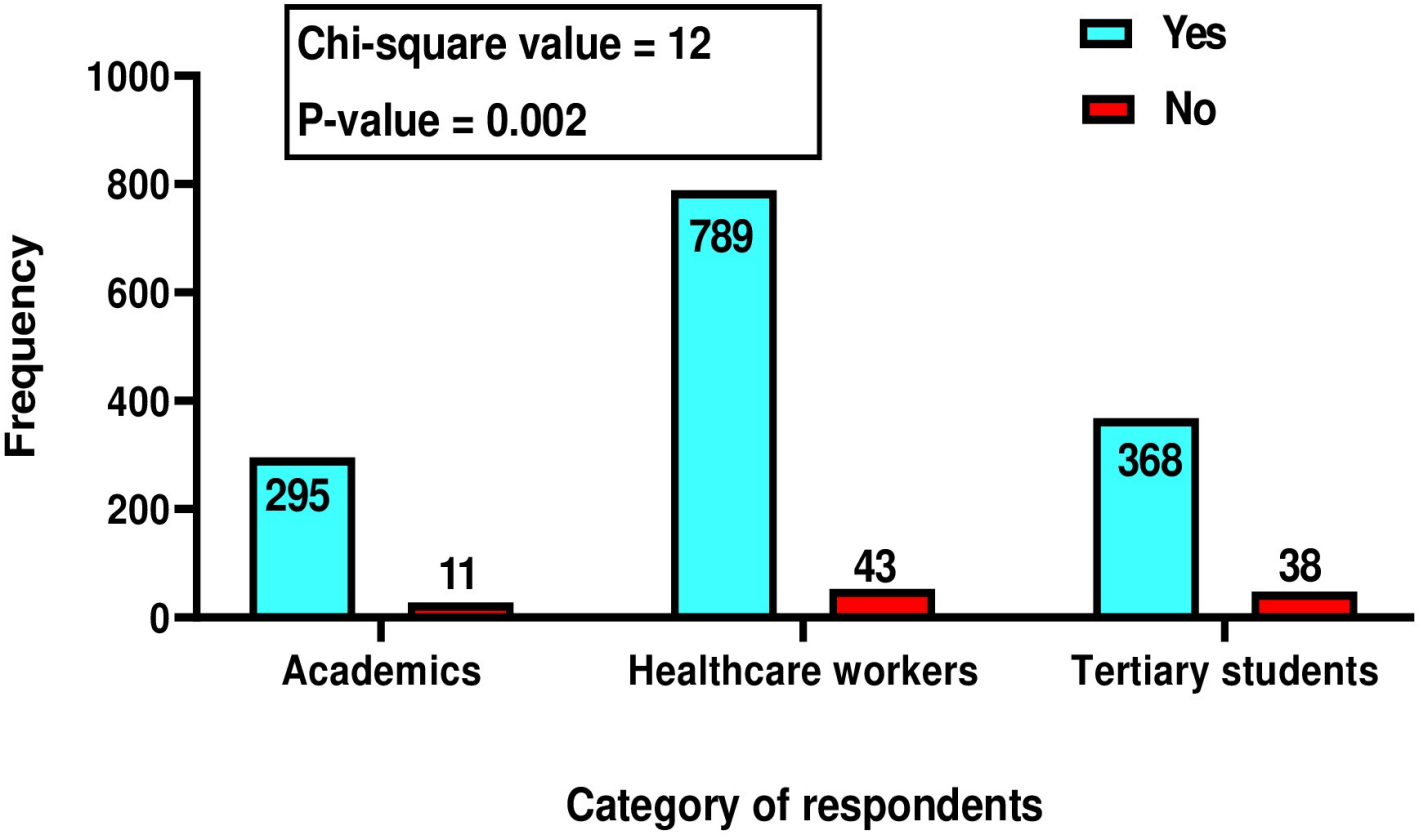
Association between respondents’ occupation [academics (n = 306), healthcare workers (n = 832) and tertiary students (n = 406)] and awareness (had not heard) of mpox in Nigeria.

Overall, 1,544 respondents agreed to participate in the study out of 1553 contacted. Most of the respondents were males (n = 938, 60.8%) married (n = 931, 60.3%), and had mean age of 35.2±10.9 years (Table 1). The distribution of respondents surveyed across the six geopolitical zones is presented in Fig 3. Only 92(0.1%) respondents have not heard of mpox. The tertiary students (n = 38, 41.3%) and healthcare workers (n = 43, 37%) had the highest and second largest proportion of people who never heard of the disease. Awareness about mpox was significantly associated with the occupation of respondents (Fig 4).

**Table 1:** Socio-demographic characteristics of respondents surveyed for knowledge and perception concerning mpox in Nigeria.

Variables	Frequency n = 1544	Percentage
**Age (in years)**		
<30	563	36.5
30–44	673	43.6
≥45	308	19.9
**Gender**		
Male	938	60.8
Female	606	39.2
**Highest Educational Qualification**		
Tertiary education	1418	91.8
Secondary education	126	8.2
**Occupation**		
Academics	306	19.8
Healthcare worker	832	53.9
Tertiary student	406	26.3
**Marital Status**		
Single	613	39.7
Married	931	60.3
**Geopolitical Zone of Residence**		
North-central	187	12.1
Northeast	286	18.5
North-west	335	21.7
Southeast	315	20.4
South-south	82	5.3
South-west	339	22
**Location of Residence**		
Urban Area	1267	82.1
Rural Area	277	17.9

### Perceptions of respondents about mpox

Out of 1,452 respondents that have heard of mpox, most of them knew that the disease is real (n = 1309, 90.2%), not a new (n = 903, 62.2%) and caused by a virus (n = 1320; 90.9%). Regarding the transmission, most respondents did not know that only infected monkeys, not all monkeys, can spread the disease (n = 951, 65.5%). Similarly, most respondents (n = 833, 57.4%) didn’t know that mpox can be spread by the consumptions of inadequately cooked flesh of infected animals. However, the respondents knew infected rodents could spread it (n = 983, 67.7%). Furthermore, 411 (28.3%) respondents knew that MPXV is not as highly contagious as the COVID-19 and do not spread from COVID-19 vaccine (n = 1243, 85.6%) and that the outbreak was not another plot to cause lockdown like COVID-19 did (n = 1132, 78.0%). Only 317 (28.1%) of the respondents knew that MPXV is categorized as “sexually transmitted infection” with only 306 (21.1%) feeling it is ridiculous to be worried about the infection (Table 2). There were significant differences in the responses of the three categories of respondents about perceptions towards mpox.

**Table 2:** Association between the occupation of the Nigerian respondents (academics, healthcare workers and tertiary students) and their perceptions towards the transmission, prevention and control of mpox.

Information required		Academics	Health Workers	Tertiary Students	Total	p-value
		n = 295 (%)	n = 789 (%)	n = 368 (%)	n = 1452 (%)	
Mpox is not real, It is another propaganda	Yes	14 (4.7)	24 (3.0)	16 (4.3)	54 (3.7)	<0.001
	No	266 (90.2)	732 (92.8)	311(84.5)	1309 (90.2)	
	I do not know	15 (5.1)	33 (4.2)	41 (11.1)	89 (6.1)	
Mpox is a new disease	Yes	91(30.8)	268 (34.0)	128 (34.8)	487 (33.5)	<0.001
	No	190 (64.4)	507 (64.3)	206 (56.0)	903 (62.2)	
	I do not know	14 (4.7)	14 (1.8)	34 (9.2)	62 (4.3)	
Mpox is caused by a virus	Yes	272 (92.2)	733 (92.9)	315 (85.6)	1320 (90.9)	0.001
	No	4 (1.4)	13 (1.6)	8 (2.2)	25 (1.7)	
	I do not know	19 (6.4)	43 (5.4)	45 (12.2)	107 (7.4)	
Mpox can be contracted from monkeys	Yes	199 (67.5)	555 (70.3)	197 (53.5)	951 (65.5)	<0.001
	No	50 (16.9)	145 (18.4)	59 (16.0)	254 (17.5)	
	I do not know	295 (15.6)	89 (11.3)	112 (30.4)	247 (17.0)	
Mpox is a sexually transmitted infection (STI)	Yes	61 (20.7)	171 (21.7)	85 (23.1)	317 (21.8)	<0.001
	No	184 (62.4)	536 (67.9)	193 (52.4)	913 (62.9)	
	I do not know	50 (16.9)	82 (10.4)	90 (24.5)	222 (15.3)	
Mpox spreads through contact with infected rodents	Yes	190 (64.4)	567 (71.9)	226 (61.4)	983 (67.7)	<0.001
	No	57 (19.3)	141 (17.9)	52 (14.1)	250 (17.2)	
	I do not know	48 (16.3)	81 (10.3)	90 (24.5)	219 (15.1)	
Mpox spreads by consuming inadequately cooked flesh of animals	Yes	157 (53.2)	479 (60.7)	197 (53.5)	833 (57.4)	<0.001
	No	75 (25.4)	200 (25.3)	67 (18.2)	342 (23.6)	
	I do not know	63 (21.4)	110 (13.9)	104 (28.3)	277 (19.1)	
Mpox is as highly contagious as COVID-19	Yes	161 (54.6)	502 (63.6)	216 (58.7)	879 (60.5)	<0.001
	No	103 (34.9)	225 (28.5)	83 (22.6)	411 (28.3)	
	I do not know	31 (10.5)	62 (7.9)	69 (18.8)	162 (11.2)	
Mpox was spread from COVID-19 Vaccine	Yes	6 (2.0)	12 (1.5)	17 (4.6)	35 (2.4)	<0.001
	No	246 (83.4)	712 (90.2)	285 (77.4)	1243 (85.6)	
	I do not know	43 (14.6)	65 (8.2)	66 (17.9)	174 (12.0)	
Mpox is another plot to cause lock down like COVID-19	Yes	22 (7.5)	74 (9.4)	50 (13.6)	146 (10.1)	<0.001
	No	236 (80.0)	651 (82.5)	245 (66.6)	1132 (78.0)	
	I do not know	37 (12.5)	64 (8.1)	73 (19.8)	174 (12.0)	
It is ridiculous to be worried about Mpox	Yes	50 (16.9)	174 (22.1)	82 (22.3)	306 (21.1)	0.019
	No	231 (78.3)	590 (74.8)	261 (70.9)	1082 (74.5)	
	I do not know	14 (4.7)	25 (3.2)	25 (6.8)	64 (4.4)	

Pearson’s Chi-square statistic using IBM^®^ SPSS version 20 (SPSS Inc., Chicago, Illinois, USA)

### Knowledge of mpox prevention, infection and transmission dynamics

The majority (n = 1188, 80.8%) of the respondents knew that MPXV could be transmitted through broken skin and contacts with infected bodily fluid, however, less than 60% knew it could be transmitted through respiratory droplets, contaminated clothing or linen and consumption of inadequately cooked flesh of infected animals. Most respondents (n = 1024, 70.5%) affirmed that MPXV infection could be contracted with close contact with asymptomatic people. Regarding treatment, 88.2% stated that there is treatment, while 55.4% indicated a cure for mpox is available (Table 3).

**Table 3:** Knowledge of academics, health workers and tertiary students about the transmission, symptoms prevention and control of mpox virus infections in Nigeria.

Questions asked/ information required	Responses	Academics (n = 295)	Health Worker (n = 789)	Tertiary Student (n = 368)	Total (n = 1452)	P-value
*Modes of transmission*						
Broken skin-to-skin contact with infected person	Yes	230 (78.0)	663 (84.0)	295 (80.2)	1188 (81.8)	0.045
Respiratory droplets	Yes	146 (49.5)	469 (59.4)	195 (53.0)	810 (55.8)	0.006
Mucous membranes, like the eyes, nose, and mouth	Yes	169 (57.3)	517 (65.5)	213 (57.9)	899 (61.9)	0.008
A bite or scratch from an infected animal	Yes	188 (63.7)	552 (70.0)	223 (60.6)	963 (66.3)	0.004
Contact with infected bodily fluids	Yes	230 (78.0)	657 (83.3)	280 (76.1)	1167 (80.4)	0.008
Contaminated clothing or linens	Yes	142 (48.1)	430 (54.5)	159 (43.2)	731 (50.3)	0.001
Consuming inadequately cooked flesh of infected animals	Yes	162 (54.9)	483 (61.2)	201 (54.6)	846 (58,3)	0.045
*Symptoms in humans*						
Fever	Yes	253 (85.8)	729 (92.4)	320 (87.0)	150 (10.3)	0.001
Chills	Yes	140 (47.5)	457 (57.9)	187 (50.8)	784 (54.0)	0.003
Headache	Yes	208 (70.5)	614 (77.8)	260 (70.7)	1082 (74.5)	0.007
Fatigue	Yes	181 (61.4)	552 (70.0)	216 (58.7)	949 (65.4)	<0.001
Swollen lymph nodes	Yes	178 (60.3)	579 (73.4)	218 (59.2)	975 (67.1)	<0.001
Rash	Yes	215 (72.9)	638 (80.9)	251 (68.2)	1104 (76.0)	<0.001
Blisters	Yes	189 (64.1)	503 (63.8)	193 (52.4)	885 (61.0)	0.001
Sores in mouth	Yes	125 (42.4)	411 (52.1)	157 (42.7)	693 (47.7)	0.001
Sores in vagina	Yes	85 (28.8)	225 (28.5)	86 (23.4)	396 (27.3)	0.150
Sores in anus	Yes	80 (27.1)	229 (29.0)	82 (22.3)	391 (26.9)	0.055
No Symptoms	Yes	8 (2.7)	30 (3.8)	18 (4.9)	56 (3.9)	0.348
*Prevention and control methods*						
Mpox is curable	Yes	163 (55.3)	468 (59.3)	173 (47.0)	804 (55.4)	<0.001
	No	42 (4.2)	102 (12.9)	35 (9.5)	179 (12.3)	
	I do not know	90 (30.5)	219 (27.8)	160 (43.5)	469 (32.3)	
Mpox can be prevented by avoiding contact with animals suspected to have or died of Mpox	Yes	262 (88.8)	728 (92.3)	314 (85.3)	1304 (89.8)	<0.001
	No	11 (3.7)	27 (3.4)	10 (2.7)	48 (3.3)	
	I do not know	22 (7.5)	34 (4.3)	44 (12.0)	100 (6.9)	
Mpox can be prevented by thoroughly cooking of all foods of animal origin	Yes	230 (78.0)	666 (84.4)	270 (73.4)	1166 (80.3)	<0.001
	No	35 (11.9)	71 (9.0)	23 (6.2)	129 (8.9)	
	I do not know	30 (10.2)	52 (6.6)	75 (20.4)	157 (10.8)	
Mpox can be prevented by hand washing frequently with soap and water	Yes	244 (82.7)	706 (89.5)	310 (84.2)	1260 (86.8)	0.002
	No	25 (8.5)	47 (6.0)	21 (5.7)	93 (6.4)	
	I do not know	26 (8.8)	36 (4.6)	37 (10.1)	99 (6.8)	
Mpox can be prevented by practising safer sex, including the use of condoms and dental dams	Yes	141 (47.8)	467 (59.2)	210 (57.1)	818 (56.3)	<0.001
	No	92 (31.2)	215(27.2)	65 (17.7)	372 (25.6)	
	I do not know	62 (21.0)	107 (13.6)	93 (25.3)	262 (18.0)	
Mpox can be prevented by getting vaccinated against small pox	Yes	133 (45.1)	425 (53.9)	206 (56.0)	764 (52.6)	<0.001
	No	88 (29.8)	206 (26.1)	55 (14.9)	349 (24.0)	
	I do not know	74 (25.1)	158 (20.0)	107 (29.1)	339 (23.3)	
There is no treatment available for Mpox	Yes	106 (35.9)	310 (39.3)	89 (24.2)	505 (34.8)	<0.001
	No	110 (37.3)	325 (41.2)	120 (32.6)	555 (38.2)	
	I do not know	79 (26.8)	154 (19.5)	159 (43.2)	392 (27.0)	
There is an available vaccine for Mpox	Yes	106 (35.9)	276 (35.0)	127 (34.5)	509 (35.1)	<0.001
	No	96 (32.5)	308 (39.0)	76 (20.7)	480 (33.1)	
	I do not know	93 (31.5)	205 (26.0)	165 (44.8)	463 (31.9)	

Pearson’s Chi-square statistic using IBM^®^ SPSS version 20 (SPSS Inc., Chicago, Illinois, USA)

Results showed that 1167 (80.4%) of the respondents knew that MPXV infection could be prevented by avoiding contact with animals suspected to have or died the disease (n = 1304, 89.3%) and by frequent hand washing with soap and water and by cooking all foods of animal origin. However, only 509 (35.1%) of the respondents knew that vaccines are available for inoculation against mpox while 818 (56.3%) know that the disease could not be prevented by practising safer sex, including the use of condoms and dental dams (Table 3).

When assessed on symptoms of pox, the clinical symptoms recognized by most of the respondents were skin rash and headache (>70%). A majority (>70%) did not know that fever, sores in the anus and vagina are symptoms of mpox, while very few (n = 56, 3.9%) knew that asymptomatic cases are possible (Table 3). There were significant differences in the responses of the three categories of respondents on knowledge about mpoxX except two responses under clinical symptoms.

### Relationship between socio-demographics and perception and knowledge scores

Table 4 shows the relationship between socio-demographics of the respondents with their perception and knowledge scores. Perception scores ranged from 0 to 11. The mean perception score was 4.5± 2.0. Since the maximum obtainable score was 11 points, respondents with more than 5.5 (average of 11) and those with ≤ 5.5 were categorized as having positive and negative perceptions about mpox, respectively. Only 419 (28.9%) respondents had positive perception about mpox. Respondents’ characteristics that were associated with perception were highest educational qualification (p = 0.001), occupation (p = 0.029), geopolitical zone (p <0.001) and location of residency (p <0.001). Knowledge score ranged from 0 to 9 with a mean score of 5.8+1.9. Respondents with more than 5.8 scores were regarded as having adequate knowledge, while those with 5.8 and lower were regarded as having inadequate knowledge. Most of the respondents (60.5%) had adequate knowledge about mpox. Respondents’ characteristics that were associated with knowledge level were age group (p = 0.02) highest educational qualification (p = 0.004), occupation (p <0.001) and geopolitical zone of residency (p = 0.001). There was significant positive correlation between perception and knowledge scores (r = 0.4, p <0.001). Positive perceptions were more likely among respondents who had tertiary education and lived in rural areas in North-west Nigeria. Likewise, adequate knowledge scores were more likely among respondents younger than 30 years of age, had tertiary education, and resided in North-west Nigeria (Table 5).

**Table 4:** Factors associated with respondents’ perception and knowledge toward Mpox virus infection in Nigeria.

Factors	Perception	P value	Knowledge	P-value
	Positive (%*)		Adequate (%*)	
*Age (in years)*				
<30	143 (28.0)	0.121	298(58.4)	0.020
30–44	201(31.3)		413(64.3)	
≥45	75(25.0)		167(55.7)	
*Gender*				
Male	254(28.8)	0.951	539(61.1)	0.533
Female	165(28.9)		339(59.5)	
*Highest educational qualification*				
Tertiary education	403(30.1)	0.001	824(61.5)	0.004
No tertiary education	16(14.2)		54(47.8)	
*Occupation*				
Academics	70(23.7)	0.029	152(51.5)	<0.001
Healthcare worker	249(31.6)		531(67.3)	
Tertiary Student	100(27.2)		195(53.0)	
*Marital status*				
Single	154(27.4)	0.331	324(57.7)	0.081
Married	265(29.8)		554(62.2)	
*Geo-political zone of residence*				
North-central	55(32.2)	0.000	112(65.5)	0.001
Northeast	96(36.4)		145(54.9)	
North -west	118(37.6)		217(69.1)	
Southeast	72(24.0)		182(60.7)	
South-south	22(27.5)		48(60.0)	
South-west	56(17.3)		174(53.9)	
*Location of residence*				
Urban Area	299(25.0)	0.000	715(59.7)	0.184
Rural Area	120(47.2)		163(64.2)	

*Percentage of the respondents with positive perception and adequate knowledge within each level of the considered factors

**Table 5:** Multivariable logistic regression analyses of factors of perception and knowledge towards mpox virus infection in Nigeria.

Factors	Perception*	P value	Knowledge**	P value
	OR (95%CI)		OR (95%CI)	
*Age (in years)*				
<30	1.30 (0.8–2.11)	0.284	1.81(1.15–2.83)	0.010
30–44	1.19 (0.85–1.66)	0.310	1.44(1.07–1.93)	0.017
≥45	1	0.503	1	0.018
*Highest Educational Qualification*				
Tertiary education	2.50 (1.37–4.53)	0.003	1.34 (.87–2.08)	0.185
No tertiary education	1		1	
*Occupation*				
Academics	0.70 (0.44–1.11)	0.132	0.98(0.65–1.48)	0.916
Health Worker	0.90 (0.62–1.31)	0.588	1.80(1.26–2.55)	0.001
Tertiary Student	1	0.235	1	<0.001
*Marital Status*				
Single	0.89 (.612–1.28)	0.522	0.87(.61–1.23)	0.424
Married	1		1	
*Geo-political zone of residence*				
North-central	2.15 (1.37–3.37)	0.001	1.53(1.03–2.28)	0.037
Northeast	2.42 (1.62–3.59)	0.000	0.92 (0.66–1.29)	0.631
North -west	2.54 (1.72–3.76)	0.000	1.59 (1.13–2.25)	0.009
Southeast	1.32 (0.87–2.00)	0.196	1.38 (0.98–1.94)	0.064
South-south	1.47 (0.82–2.65)	0.198	1.08 (0.65–1.81)	0.758
South-west	1	0.000	1	0.011
*Location of residence*				
Urban Area	0.38 (0.29–0.51)	0.000	0.86(0.64–1.15)	0.304
Rural Area	1		1	

*Hosmer-Lemeshow goodness of fit test statistic = 4.233, P-value = 0.855

**Hosmer-Lemeshow goodness of fit test statistic = 13.887, P-value = 0.085

### Sources of information on mpox

**Fig 5:**
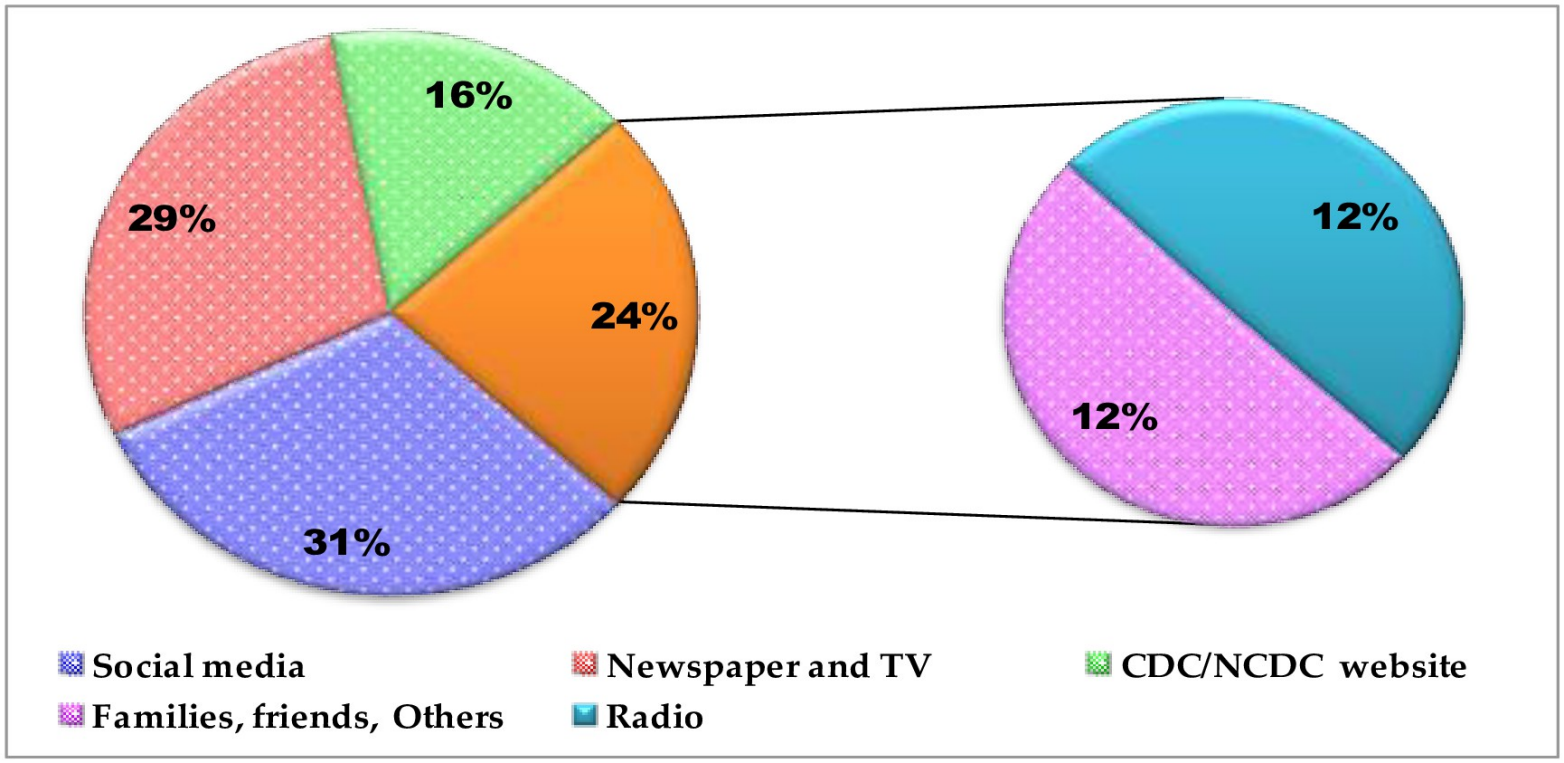
Various sources of information on mpox virus infection among academics, healthcare workers and tertiary students surveyed in Nigeria. CDC = Centers for Disease Control and Prevention; NCDC = Nigeria Centre for Disease Control.

Fig 5 shows the reported sources of information by the respondents. Social media was the most frequently reported source of information (31.1%) followed by newspaper and TV (29.0%), while the least was radio (11.7%). Respondents with the highest percentage of positive perception and adequate knowledge sourced information from family, friends and others and CDC/NCDC websites respectively. Sources of information also significantly affected both the perception and knowledge of the respondents (Table 6).

**Table 6:** Relationship between respondents’ perception and knowledge scores in relation to sources of information about mpox virus infection in Nigeria.

Sources of Information	Count n = 3134 (%)	Valid (%)	Perception score		P-value	Knowledge score		P-value
			Negative (%)	Positive (%)		Inadequate (%)	Adequate (%)	
CDC/NCDC websites	512(35.3)	16.3	342(66.8)	170(33.2)	0.004	123(24.0)	389(76.0)	<0.001
Newspaper and TV	908 (62.5)	29.0	668(73.6)	240(26.4)		343(37.8)	565(62.2)	
Radio	368(25.3)	11.7	248(67.4)	120(32.6)		112(30.4)	256(69.8)	
Social media	974(67.1)	31.1	687(70.5)	287(29.5)		371(38.1)	603(61.9)	
Families, friends and others	372(25.6)	11.9	238(64.0)	134(36.0)		130(34.9)	242(65.1)	

CDC = Centers for Disease Control and Prevention; NCDC = Nigeria Centre for Disease Control

## Discussion

The results which indicate that most respondents had tertiary education (91.8%) and belonged to the young (≤ 44 years old) population (79.1%) may help for better management and control of mpox in Nigeria. This is because the findings suggest that these critical stakeholders (respondents) may possess the requisite mental and physical ability, to combat MPXV infection in Nigeria. A young and highly educated workforce will likely possess and deploy both the physical strength/resilience as well as the intellectual capability/skill requisite to fight endemic/re-emerging infections like MPXV [33]. The findings imply that if adequately mobilised, the Nigeria’s health and educational sectors, can gallantly fight to control and possibly eradicate MPXV infection in the country; in collaboration with other relevant sectors and agencies. Inadequate funding and lack of modern health and research facilities had been the bane of these critical sectors; and had unabatedly limited the potentials of the personnel. Therefore, adequate and timeous funding, as well as provision of modern health-related research facilities is crucial; for harnessing the capabilities of the Nigerian health and academic personnel towards the control and possible eradication of MPXV and other endemic infectious diseases.

However, 43 (5.2%) healthcare workers had not heard of mpox (Fig 4). Although this category of respondents seems small, it is very significant from public health and global health standpoints. Admitting that they have not heard of mpox implies that they are oblivious to the disease and will likely not include MPXV infection in their differential diagnosis of patients presenting with symptoms suggestive of the disease. When a disease that has attained the status of PHEIC is misdiagnosed, then mistreatment or mismanagement of the case is most probable. This may lead to further dissemination or even case exportation of the infection, all at the detriment of public and global health. This lack of awareness calls for more enlightenment campaigns on MPXV infections among healthcare workers. This could be achieved by emphasising emerging and re-emerging zoonoses during the routine compulsory professional continuing education among health professionals.

The finding that 60.5% of the respondents had adequate knowledge of the modes of transmission, clinical symptoms and methods of prevention and control of MPXV infection (Table 3) is quite interesting. This overall good knowledge is not unexpected considering that the categories of the respondents in this study are among the best brains in the society and are in a better position to understand the symptomatology of the disease as well as the prevention and control measures. The good knowledge found presupposes that the respondents may not only recognise the symptoms suggestive of mpox but may also take appropriate measures to protect themselves from getting infected or spreading the disease. Despite the overall good knowledge, 62% and 51.5% of academics didn’t know that MPXV is transmissible via contaminated clothing and infectious droplets respectively. These call for massive enlightenment campaign on the transmissibility of the virus through inhalation of infectious droplets and skin contact with infectious fomites contaminated with MPXV, particularly among academics. Since, the social media, newspaper and TV are the commonest sources of information as found in this study; these mass media could be used for the enlightenment campaign. However, there is need for caution on the use of social medial for mass education due to infodemic and other misinformation associated with social media [34].

The 60.5% adequate knowledge found in this study is lower than the 70% good knowledge found among Jordanian healthcare students [35]. However, the 60.5% adequate knowledge is higher than 48% among the general population in Saudi Arabia [32], 36.6% in Indonesian general healthcare practitioners [36], 27% among Italian medical professionals [37] and 47% found among the general public in the US [38]. Similarly, the overall knowledge score of 4.8 found in this study is higher than 3.8 reported in Kuwait [39]. The disparity in the findings could be due to discrepancies in the population sample size, endemicity of MPXV infection in the countries and category of respondents. In this study, a large sample size of 1,544 respondents was surveyed unlike in the other studies. A low sample size could introduce bias and inaccuracies in the findings [40]. Considering that MPXV was predominantly circulating in African continent before the first case exportation to the US in 2003 [15], it is likely that the general population and the medical professionals in the mpox non-endemic countries may not have much information about the disease. In both mpox endemic and non-endemic countries, it is also expected that healthcare workers (due to their training), unlike the general population, should have a better knowledge of MPXV, including the transmission dynamics and prevention/control strategies for the infection. These may explain the dichotomy in the findings regarding knowledge of mpox among the various populations and countries reported above.

Notwithstanding the overall good/adequate knowledge found in this work, it is noteworthy that the knowledge did not translate to positive perception (Tables 4 and 5) in all the factors of interest (age, gender, occupation, educational qualification, geopolitical zone, marital status and location of residence) studies. For instance, 61.5% of respondents who had tertiary education had good knowledge on MPXV transmission, symptoms and prevention/control but only 30.1% had positive perceptions about the disease. Similarly, 67.5% of the healthcare workers had good knowledge of mpox virus transmission, symptoms and prevention/control but only 31.6% had positive perception about the disease. The perception about mpox found in this study agrees with the report of Ghazy *et al*. [41] in which 58.4% of Nigerian health workers expressed complacency towards mpox vaccination. Although it is difficult to predict the exact reason for the mismatch between good knowledge and positive perception; it may not be unconnected with the facts that MPXV infection is a self-limiting disease and the West African clade largely responsible for the disease in Nigeria has a lower case fatality ratio [22]. It has been reported that the case fatality ratio of the West African MPXV variant predominant in Nigeria is low (3.6%) compared to 10.6% reported for the more virulent Central African strain circulating in Central African countries [4]. The low case fatality ratio and the self-limiting nature of the virus may have elicited complacent mentality and attitude among the respondents, hence the misalliance between good knowledge and positive perception noted in the study. Should this be the case, there is need for caution among the respondents and indeed the general population because of the paradigm shift in MPXV epidemiology in which the highly virulent clade has been found in West African country and cases exportation to other non-endemic continents have been reported [4, 14, 15, 22].

To effectively control MPXV infection at the global stage, there is a need to liberalise the availability of the new vaccine against mpox called MVA-BN (Modified Vaccinia Ankara—Bavarian Nordic). This vaccine is the only one approved by the WHO in 2019 for inoculation against the disease. Global inequity in access to vaccines and therapeutics against infectious diseases had continued to be a major challenge in the fight against infectious disease of global health importance [42]. High-income countries have been accused of stockpiling and administering the only available mpox vaccines, while low income countries have little or no access to the vaccine [43]. Unfortunately, the cessation of production and administration of smallpox vaccination in developing countries, which provided about 85% cross-protection against mpox, may have increased the virus transmission [4, 6]. The case exportation of MPXV outside the traditionally endemic African counties highlights the global relevance of the disease. It underscores the need to prioritize the mpox endemic Central and West African countries, especially Nigeria, in the distribution of the vaccine and other antiviral therapeutics so far approved for the management of the disease. Cognizant that WHO does not recommend mass vaccination against mpox but only recommends cluster vaccination in endemic areas, case contacts, healthcare workers and other at-risk-individual, particularly in the traditionally endemic African countries [22]; there is absolutely no need to stockpile the vaccine. Instead, there should be equitable distribution of the available vaccine such that mpox endemic counties, having higher burden of the infection/disease, should receive higher share of the available vaccine; to reduce the in-country disease transmission, and case exportation for global health safety. Lack of the vaccine in endemic African regions may perpetuate the zoonotic transmission of the MPXV; enhance the human-to-human transmission or even the reverse zoonotic spread. Already, human to dog transmission has been reported [18]. This could aid the virus adaptation or mutation in new hosts, jumping of specie levels or emergence of more virulent variants.

Additionally, there is serious concern regarding the spread of this virus from humans to animals, including wildlife and pets, as this could result in the establishment of new reservoir host populations, which could make the virus endemic in traditionally non-endemic regions and worsen the global public health problem caused by mpox [44–46]. The establishment of new reservoir hosts of MPXV is major setback to the control and possible eradication of mpox as the situation could worsen both public health and animal health at the global stage. Consequently, the risk of human-to-animal spillback, which is enormous due to human-animal bonding especially with pets and non-human primates, needs to be urgently addressed to avert mpox pandemic or epizootic. This could be achieved through responsible pet ownership, efficient medical/veterinary waste disposal and increased precautionary and preventive measures, in places where animals live in close proximity with mpox infected individuals.

The negative perception about mpox found among the respondents, who are the “crème de lacreme” in the Nigerian society and are in the best position to flatten the epidemiological curve of the disease, is a global public health risk. This is because the necessary steps and basic precautionary measure needed to limit the transmission of the diseases may be jettisoned, leading to increased national spread and hence massive global case exportation of MPXV from endemic countries. With increased international travel due to the removal of almost all COVID-19 restrictions globally [43, 47], the international spread of MPXV, especially from traditionally endemic countries may be inevitable. Considering the rising level of mpox in Nigeria, her fragile health sector and high level of international travel between Nigeria and other countries, especially in the American, European and Asian continents; case exportation of the virus to these countries cannot be ruled out. Further global spread of the exported MPXV to both human and animal population in other non-endemic countries is most likely considering the high infectivity of the virus, the high level of various kinds of socialization and human-animal bounding in most Non-African countries [46]. In view if the global public health importance of the rising cases of mpox in Nigeria, there is an urgent need for effective control of the infection through awareness creation to improve positive perception of the disease, and support of the countries health sector for better diagnosis and prevention of the disease. To this end, One Health control approach, involving animal and human health workers, is imperative for active surveillance against MPXV in reservoir hosts (rodents and non-human primates) and for prevention of reverse zoonotic transmission of the virus at the human-animal interface. Di- Gennaro et al. [45] recommend that such surveillance be carried out using the polymerase chain reaction (PCR) test is the gold standard laboratory test. Research collaboration and technology transfer for mass production of available mpox vaccines and drugs, as well as the development of new ones may be worthwhile, even though Harapan et al. and Ophinni et al. [48, 49] suggested that MPXV can invade both innate immunity and protection conferred by the modified vaccinia virus Ankara. In the interim, prioritization of mpox endemic African countries, especially Nigeria, in the distributions of vaccines for targeted vaccination against mpox and approved drugs for prompt management of the disease may limit the case exportation and hence safeguard global public health.

## Conclusion

The findings of this study generally reveal high knowledge of mpox and negative perception of the disease as compared with standard statistical thresholds. Knowledge and perceptions were positively influenced by age, academic qualification, occupation and geographical zone of residence. Since mpox is endemic in Nigeria and the knowledge and perceptions of healthcare workers, academics and tertiary students towards the disease may be critical epidemiological determinants of its transmission rate, it is imperative that intensive awareness campaigns be targeted at aforementioned demographic strata. This could be achieved through different media, particularly social media and Newspaper/TV news, to increase knowledge and more importantly influence perception positively. This could safeguard public health by significantly reducing the dissemination of MPXV infection in Nigeria and the possible case exportation, especially to the non-endemic global community.

### Strengths and limitations of the study

This is the first nation-wide study to investigate perception and knowledge of mpox among healthcare workers, academics and tertiary students in Nigeria, with a view to explain the rising cases of the infection in the country. The major results point to poor perception and inadequate knowledge of the disease transmission dynamics among healthcare workers, academics (researchers) and tertiary students. Furthermore, the results highlight the importance of social media as the respondents’ preferred sources of information regarding mpox.

The study however has some unavoidable limitations which included the use of cross sectional study design which did not take into consideration changes in the knowledge of the participants in relation with time. Also, sampling was not randomized which could have introduced bias into the study. Moreover being an online survey it is possible that some targeted population could have been excluded. Finally, self-reported nature of questionnaire survey is subject to information manipulation.

## Supporting information

S1 Table A copy of the questionnaire used in this study. (DOCX)

S2 Table Output of the Hosmer-Lemeshow test used to determine the goodness of fit of the statistical model used in this study. (DOC)
