# Supplementary material for: Mpox in Nigeria: Perceptions and knowledge of the disease among critical stakeholders—Global public health consequences
Source: PLoS One. 2023 Mar 30;18(3):e0283571. doi: 10.1371/journal.pone.0283571 (PMC10062623; doi:10.1371/journal.pone.0283571)
Supplement: S1 Table — (DOCX) [file pone.0283571.s001.docx]

**Knowledge and Perception of Nigerian Healthcare workers, Academics and Tertiary students towards Monkeypox**

Dear Respondent, This survey aims at gathering data on Knowledge and Perception of Nigerian Healthcare workers, Academics and Tertiary students towards Monkeypox. Data to be gathered will be confidentially treated and solely used for research purposes. **Only individuals who are 18 years and above and belong to any of the three category of respondents are kindly requested to participate in the study.**

Would you like to voluntarily participate in the survey and provide responses to the under-listed questions?

- I agree to participate
- Decline

## **Socio-demographics**

1. Gender

- Male
- Female

1. Age (as at last birthday in years)
2. Highest Educational Qualification

- Primary education
- Secondary education
- Tertiary education

1. Occupation

- Tertiary Student
- Academics
- Healthcare worker

1. Marital Status

- Single
- Married

1. Geo-political Zone of residence

- North-Central (NC)
- Northeast (NE)
- North-West (NW)
- South-West (SW)
- Southeast (SE)
- South-South (SS)

1. Location of Residence

- Rural Area
- Urban Area

## **Knowledge and Misconceptions on Monkeypox**

1. Have you heard of Monkeypox

- Yes
- No

| Please respond to the statements below | Yes | No | I don’t know |
| --- | --- | --- | --- |
| 1. Monkeypox is not real, It is another propaganda |  |  |  |
| 1. Monkeypox is a new disease |  |  |  |
| 1. Monkeypox is caused by a virus |  |  |  |
| 1. Monkeypox can be contracted from monkeys only |  |  |  |
| 1. Monkeypox is a sexually transmitted infection (STI) |  |  |  |
| 1. Monkeypox spreads through contact with infected rodents |  |  |  |
| 1. Monkeypox spreads by consuming inadequately cooked flesh of animals |  |  |  |
| 1. Monkeypox is as highly contagious as COVID-19 |  |  |  |
| 1. Monkeypox was spread from COVID-19 Vaccine |  |  |  |
| 1. Monkeypox is another plot to cause lock down like COVID-19 |  |  |  |
| 1. It is ridiculous to be worried about Monkeypox |  |  |  |

## **Knowledge on modes of transmission and symptoms of Monkeypox**

1. Monkeypox can be spread through? (choose as many as are correct)

- Broken skin to skin contact with infected person
- Respiratory droplets
- Mucous membranes, like the eyes, nose, and mouth
- A bite or scratch from an infected animal
- Contact with infected bodily fluids
- Contaminated clothing or linens
- Consuming inadequately cooked flesh of animals

1. Select appropriate symptoms of Monkeypox (choose as many as are correct)

- Fever
- Chills
- Headache
- Fatigue
- Swollen lymph nodes
- Rash
- Blisters
- Sores in mouth
- Sores in vagina
- Sores in anus
- No Symptoms

1. Which of these symptoms have you experienced within the last 6 months?(choose as many as are correct)

- Fever
- Chills
- Headache
- Fatigue
- Swollen lymph nodes
- Rash
- Blisters
- Sores in mouth
- Sores in vagina
- Sores in anus
- No Symptoms

1. An infected person without symptoms, can infect others in close contact?

- Yes
- No
- Not Sure

1. Is Monkeypox curable?

- Yes
- No
- Not Sure

## **Knowledge on prevention and control of Monkeypox**

1. Monkeypox can be prevented by avoiding contact with animals suspected to have or died of Monkeypox?

- Yes
- No
- I don’t know

1. Monkeypox can be prevented by thoroughly cooking all foods of animal origin?

- Yes
- No
- I don’t know

1. Monkeypox can be prevented by handwashing frequently with soap and water?

- Yes
- No
- I don’t know

1. Monkeypox can be prevented by practicing safer sex, including the use of condoms and dental dams?

- Yes
- No
- I don't know

1. Monkeypox can be prevented by getting vaccinated against smallpox?

- Yes
- No
- I don't know

1. No treatment available for Monkeypox

- Yes
- No
- I don't know

1. There is an available vaccine for Monkeypox

- Yes
- No
- I don't know

1. Where do you get your information updates on Monkeypox (Tick as many that apply)

- CDC/NCDC websites
- News
- Radio
- Social Media
- Family, Friends and others
