# Supplementary material for: Mpox in Nigeria: Perceptions and knowledge of the disease among critical stakeholders—Global public health consequences
Source: PLoS One. 2023 Mar 30;18(3):e0283571. doi: 10.1371/journal.pone.0283571 (PMC10062623; doi:10.1371/journal.pone.0283571)
Supplement: S2 Table — (DOC) [file pone.0283571.s002.doc]

Table S2: Hosmer-Lemeshow test output for perception and knowledge to show the goodness of fit of the models used in this study

| | **Hosmer and Lemeshow Test for Perception** | | | | | --- | --- | --- | --- | | Step | Chi-square | df | Sig. | | 1 | 4.233 | 8 | .835 |   **Hosmer and Lemeshow Test for Knowledge** | | | |
| --- | --- | --- | --- | --- | --- | --- | --- | --- | --- | --- | --- | --- | --- | --- | --- |
| Step | Chi-square | df | Sig. |
| 1 | 13.887 | 8 | .085 |
